# Assessment of Bidirectional Relationships between Mental Illness and Rheumatoid Arthritis: A Two-Sample Mendelian Randomization Study

**DOI:** 10.3390/jcm12030944

**Published:** 2023-01-25

**Authors:** Shate Xiang, Rongyun Wang, Lijiangshan Hua, Jie Song, Suhai Qian, Yibo Jin, Bingyue Zhang, Xinghong Ding

**Affiliations:** 1 School of Basic Medical Sciences, Zhejiang Chinese Medical University, Hangzhou 310053, China; 2 School of Nursing, Zhejiang Chinese Medical University, Hangzhou 310053, China; 3 School of Public Health, Zhejiang Chinese Medical University, Hangzhou 310053, China

**Keywords:** mendelian randomization, bipolar disorder, broad depression, major depression, anxiety, rheumatoid arthritis

## Abstract

A correlation between mental illness and systemic rheumatoid arthritis (RA) has been observed in several prior investigations. However, little is known about the causative relationship between them. The present study aimed to systematically investigate the potential association between genetically determined mental illness and RA. Two-sample bidirectional Mendelian randomization (MR) analysis was performed using publicly released genome-wide association studies (GWAS). We selected independent genetic variants associated with four mental illnesses (bipolar disorder, broad depression, major depression, and anxiety) as instrumental variables. The inverse variance weighted (IVW) method was used as the primary analysis to assess the causal relationship between mental illness and RA. Results of the IVW analysis suggested that genetic predisposition to bipolar disorder was associated with a decreased risk of RA (odds ratio [OR] = 0.825, 95% CI = 0.716 to 0.95, *p* = 0.007). Furthermore, we did not find a significant causal effect of RA on bipolar disorder in the reverse MR analysis (*p* > 0.05). In addition, our study found no evidence of a bidirectional causal relationship between genetically predicted broad depression, major depression, anxiety, and RA (*p* > 0.05). The genetically proxied bipolar disorder population has a lower RA risk, which may indicate that there is a hidden mechanism for inhibiting the pathogenesis of RA in bipolar disorder. However, results do not support a causal connection between depression, anxiety, and RA.

## 1. Introduction

Rheumatoid arthritis (RA) is a chronic systemic inflammatory disease, which mainly accumulates in hand and foot joints, resulting in cartilage and bone damage and disability [1]. The prevalence of RA has been increasing since 1990 [2]. Exploring the risk factors that may lead to RA-onset is still an active research field.

Mental illness, including bipolar disorder, depression, and anxiety [3,4], is one of the most important causes of death globally [5]. It was reported that mental illness is one of the most common complications in patients with RA and there is a significant correlation between them. Among them, the incidence of RA in patients with depression increased [6,7]. At the same time, the incidence of depression was 38.8% in RA patients, while the prevalence of major depression was 16.8% [8]. Cross-trait polygenic risk score (PRS) analysis supported their bidirectional association [9]. In addition, the rate of anxiety in RA patients ranges from 13 to 70% [10,11]. 

However, the occurrence rate of bipolar disorder in patients with RA remains controversial. Several studies support that the prevalence of bipolar disorder in RA patients is higher than that in non-RA patients [12,13]. The prevalence of bipolar disorder in RA patients is much lower than that in RA patients with major depression [14]. However, multivariate analysis indicated that the correlation between RA and bipolar disorder was not present [15].

Mental illness leads to increased disease activity, further deterioration of dysfunction, and decreased quality of life, and may reduce susceptibility to first-line antirheumatic drugs in patients with RA [16,17,18]. Therefore, exploring whether mental illness is a risk factor for RA might contribute to studying RA’s pathogenesis and developing novel control strategies.

However, correlation does not imply causation. Correlation is only expressed as the statistical association between two variables, referring to quantifiable relationships in the data [19]. Correlation studies do not rule out a reverse causal relationship between two disorders. For example, RA may occur secondary to anxiety disorder or bipolar disorder. At the same time, the results of a correlation analysis cannot rule out the residual confounding factors between mental illness and RA. For example, poor physical function [20], severe synovitis [20], incident illness and chronic medical adversity [21], pain [22,23,24], sleep disorders [25,26], fatigue [25], and smoking [15] may be confounding factors that affect the outcome, because they may all lead to mental illness in patients with RA. Causality, on the other hand, is a directional relationship that manifests itself as dependence or partial dependence of the result on the cause, or as the cause causing or changing the occurrence of the result [27]. To date, a causal relationship between mental illness and RA has not been established [28].

Mendelian randomization (MR) is a statistical method that uses genetic variation (e.g., single nucleotide polymorphism (SNP) as an instrumental variable (IV) to determine causal associations between risk factors and outcome variables [29]. Since the core of MR utilizes Mendel’s second law (the law of free association), it can be considered as a randomized controlled experiment based on Mendel’s second law. At the same time, because of the constancy of the random transmission of genetic variation, it can overcome errors in results caused by confounding factors in traditional observational study designs and reduce the interference of reverse causality between exposure factors and outcome variables [30]. Therefore, this study will explore whether mental illness has a causal effect on the onset of RA and whether RA is a risk factor for mental illness through the two samples during MR analysis.

## 2. Methods

### 2.1. Study Design

**Figure 1:**
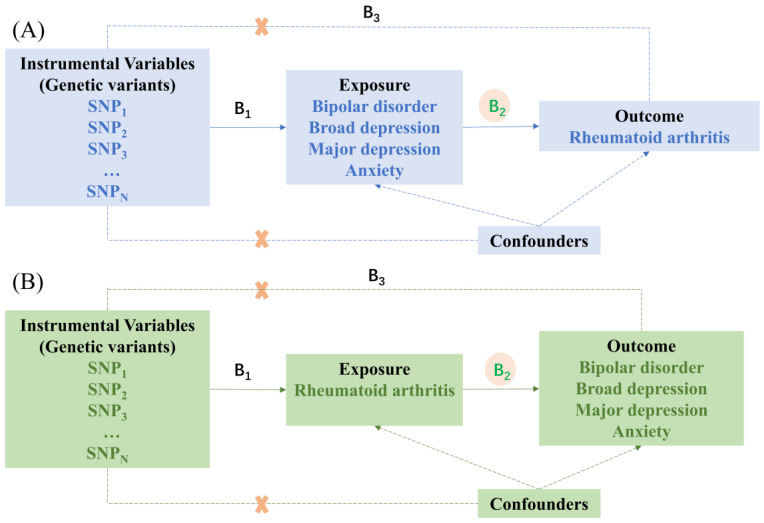
The design of MR analysis was to explore the causal effects between mental illness and rheumatoid arthritis. (**A**). The instrumental variables (IVs) were multiple single-nucleotide polymorphisms (SNP) linked to the mental disorders, the risk factors were bipolar disorder, broad depression, major depression, and anxiety, and the outcome variable was rheumatoid arthritis (RA). (**B**). The IVs are SNPs linked to the RA, the exposure factor was RA, and the outcome variables were the MR model of bipolar disorder, broad depression, major depression, and anxiety. B2 indicates the causal relationship between exposure and outcome, and B2 = B1/B3. B1 and B3 indicate the predicted value of a genetic variant on the exposure and outcome.

An overview of the study design is shown in Figure 1. The first step was to conduct a 2-sample MR analysis considering bipolar disorder, broad depression, major depression, and anxiety as risk factors and RA as the outcome (Figure 1A). The second step was to explore the causal relationship regarding RA as an exposure factor, bipolar disorder, broad depression, major depression, and anxiety as outcomes (Figure 1B).

### 2.2. Selection of Instrumental Variables

The IVs used in MR analysis need to satisfy 3 assumptions: (1) there is a strong correlation between IVs and risk factors; (2) there is no relationship between IVs and confounders and they are independent of each other; (3) there is no direct relationship between IVs and outcome variables [31].

SNPs for risk factors were derived from multiple large meta-analyses of genome-wide association studies (GWAS), which all met the permissive threshold for independent clustering of SNPs (*p* < 5 × 10^−8^) as IVs. There are 64 genomic loci associated with bipolar disorder [32], 14 loci in broad depression [33], 102 loci in major depression [34], and 10 loci in anxiety [35] (Tables S10—S13). 

Since the genomic loci of RA were the results of cross-ethnic analysis [36], we used loci that satisfy the threshold *p*  < 5 × 10^−8^ among SNPs associated with RA from European individuals. This restriction allows the study population to be limited to the European population, avoiding the frequency of genetic variation and distribution of exposure in different ethnic groups that might lead to false associations between exposure and variables.

In the MR analysis, we first ensured that the IVs were independent of each other and excluded SNPs in linkage disequilibrium (LD) by pruning SNPs within a 10,000-kb window with an r2 < 0.001 threshold. We obtained 46 genomic loci associated with RA (Table S14). In this study, the proxy SNP was not used. IVs were selected by removing palindromic SNPs with middle allele frequency (MAF). Palindromic SNPs are those with the A/T or G/C allele, whereas the MAF is from 0.01 to 0.30 [37]. Secondly, we excluded SNP related to outcome variables from the PhenoScanner (http://www.phenoscanner.medschl.cam.ac.uk/ accessed on 29 December 2022) (PhenoScanner is a database planned by the production team funded by several committees in the UK, which holds publicly available results from large-scale genome-wide association studies) (r^2^ > 0.80). Finally, we ensured that the F statistic of all IVs was greater than the threshold 10 and removed SNPs with F values less than 10 to minimize weak instrument bias [38]. The F statistic was calculated as F = R^2^ (n − k − 1)/[k (1 − R^2^)] [38]. The F-value statistics for all IVs are shown in Table S2. 

### 2.3. Data Sources

Summary statistics on broad depression, bipolar disorder, major depression, anxiety, and RA were obtained from the largest publicly available GWASs. There are clear screening criteria for the determination of each phenotype (Table 1). 

The summary statistics for bipolar disorder involved 41,917 cases and 371,549 controls from 57 cohorts (collected in Europe, North America, and Australia). The broad depression GWAS summary statistics were extracted from the UK Biobank, which analyzed 322,580 subjects (113,769 cases and 208,811 controls). Genetic variants associated with major depression were obtained from the 3 largest genome-wide depression association studies with 807,553 individuals, including personal genetics company 23 andMe, Inc., UK Biobank, and a meta-analysis of 35 cohorts (246,363 cases and 561,190 controls). Genetic variants associated with anxiety came from 9 samples of European ancestry from 7 large independent studies. To identify genetic variants contributing to genetic susceptibility shared across interview-generated, DSM-based anxiety disorders, 2 phenotypic approaches were applied: case-control (CC) comparisons and quantitative factor scores (FS). The results showed that the FS phenotype identified a larger number of associated SNPs than the CC model. Therefore, we finally used the results from the FS model as the GWAS needed in this study. The summary statistics for RA were obtained from assessments of more than 50,000 subjects, including 14,361 RA cases and 43,923 controls from 18 studies of Europeans.

The dataset used in our study was publicly available and each GWAS involved was ethically approved by the respective institutions. In addition, we checked the populations of the exposure and the outcome sample to exclude possible bias in the study results caused by sample overlap.

### 2.4. MR Analysis

MR analyses were conducted in the R statistical computing environment using the “TwoSampleMR” package. The software package can harmonize exposure and outcome data sets, including information on SNPs, effect size, standard error (se), effect allele frequency, and *p* value [40]. The Harmonise_data function was used to keep the alleles coded in the same direction. We set the minimum allele frequency to 0.01.

We used the inverse variance–weighted (IVW) method as the main causal evaluation method, which uses asymptotic estimates of the standard error of the causal (ratio) estimate of each variable. Its main characteristic is that the existence of the intercept term is not considered and the reciprocal of the local variance (the quadratic power of se) was used as the weight for fitting [41]. According to the results of heterogeneity, different models are used for IVW. When the heterogeneity is large (*p* > 0.05), a random effects model will be used to combine the effects, otherwise, a fixed effects model will be used [42].

However, this method does not take into account the uncertainty of genetic association and risk factors, and there may be a risk of underestimating the real changes in the estimates, especially when the IV is weak [41,43]. Therefore, we jointly use MR-Egger regression, weighted median approach, simple mode, and weighted mode methods as complementary methods for comparison. The weighted median approach is a further complement to the above 2 statistical methods, which is used to combine the data of multiple genetic variations into a single causal estimate, reducing the estimator error caused by invalid IVs [44]. Simple mode provides robustness for pleiotropy [45]. Weighted mode method aggregates SNPs with similar causal effects into a subset by clustering to evaluate the causal effects of a subset with a larger number of SNPs [46]. MR-Egger regression takes into account the existence of intercept terms, so it can perform weighted linear regression in the case of invalid genetic IVs, resulting in causal estimates [47].

### 2.5. Statistical Analysis

We also used Cochrane Q statistic and MR-Egger regression analysis to evaluate the bias caused by gene polymorphism. If an SNP works not only through the hypothetical approach but also through other means, it represents the existence of horizontal pleiotropy between the 2. The existence of horizontal multiplicity violates the assumption that instrumental variables are satisfied [46]. The assumption of no horizontal pleiotropy can also ensure that there is no significant deviation in the results of the IVW. In addition, Cochran’s Q was used to detect heterogeneity between different genetic instruments, excluding the impact of SNPs from different experiments and populations on the results [48].

The “leave-one-out” method was used to evaluate the stability of the results, that is, each SNP was omitted to determine whether a single SNP supports causal correlation. We also applied MR-PRESSO (Pleiotropy Residual Sum and Outlier) to detect and correct outliers that could contribute to pleiotropic bias [49]. Since our study has been tested 4 times, Bonferroni’s multiple-comparison test was considered. A *p*-value < 0.0125 meets the Bonferroni threshold of statistical significance (0.05/4 = 0.0125). Results with *p*-values greater than 0.0125 but less than 0.05 were considered suggestive evidence. Otherwise, the results were not considered statistically significant.

Finally, we retrieved potential secondary phenotypes for each SNP used as IVs from PhenoScanner (r^2^ > 0.80). The 2-sample MR study was re-evaluated after removing SNPs that may control for confounding traits to support the stability of the results.

## 3. Results

### 3.1. Causal Effect of Bipolar Disorder on RA

**Figure 2:**
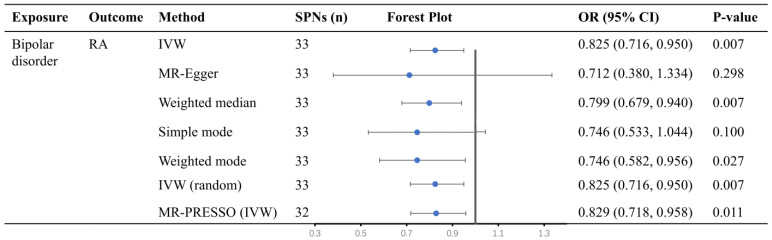
Causal relationships between bipolar disorder oandn rheumatoid arthritis by Mendelian randomization (MR) analysis.

We found evidence of a protective causal relationship between bipolar disorder and RA (IVW odds ratio [OR] = 0.825, 95% CI = 0.716 to 0.95, *p* = 0.007, Figure 2), and the result did strictly meet the multiple comparisons criterion. This result was broadly consistent with estimates from the weighted median (OR = 0.799, 95% CI = 0.679 to 0.940, *p* = 0.007, Figure 2) and weighted mode analyses (OR = 0.746, 95% CI = 0.582 to 0.956, *p* = 0.027, Figure 2). No evidence of horizontal pleiotropy was found in the Egger intercept test (*p* of Egger-intercept = 0.64; Table S1). Removal of any single instrument SNP did not appear to change the significance of the overall MR estimate (Figure S1a).

RA, rheumatoid arthritis; OR, odds ratio; CI, confidence interval; IVW (random), random-effects inverse variance weighted; MR-PRESSO, MR-pleiotropy residual sum, and outlier.

Corrected results did not change after removing one outlier using MR-PRESSO (Figure 2). In the 33 reported SNPs linked to bipolar disorder, after removing the SNP associated with potential confounding factors (Table S3), the result still showed that bipolar disorder was protective against the risk of RA (IVW OR = 0.842, 95%CI = 0.723 to 0.981, *p* = 0.03, Table S4). Nevertheless, this positive result was suggestive evidence after using the Bonferroni correction (0.0125 < *p* < 0.05). 

### 3.2. Causal Effect of Broad Depression, Major Depression, and Anxiety on RA

In the IVW analysis, we did not find any significant causal relations between broad depression (OR = 11.76, 95% CI = 0.23 to 600.62, *p* = 0.22, Table 2), major depression (OR = 1.14, 95% CI = 0.87 to 1.50; *p* = 0.35; Table 2), anxiety (OR = 1.12, 95% CI = 0.89 to 1.40; *p* = 0.35; Table 2), and RA. MR-Egger, weighted median, simple mode, and weighted mode supported this conclusion (*p* > 0.05; Table 2).

To further verify the reliability of the above results, we performed an MR-Egger intercept and the test showed no horizontal pleiotropy (*p* of Egger-intercept > 0.05; Table S1). After excluding outlier SNPs through the MR-PRESSO global test, the results were consistent with IVW results (Table S7). After removing the SNP associated with potential confounding factors (Table S3), the results did not change substantially (Table S4). Leave-one-out analysis found that rs6030245 was a high leverage point with high influence (Figure S1d). After removing rs6030245, IVW analysis showed that there was a causal effect between anxiety and RA (OR = 1.27, 95%CI = 1.03 to 1.58, *p* = 0.03, Table S8), which means that genetically determined anxiety disorders increased the risk of RA. The result indicated that the result obtained by MR was unstable.

### 3.3. Causal Effects of RA on Bipolar Disorder, Broad Depression, Major Depression, and Anxiety 

**Figure 3:**
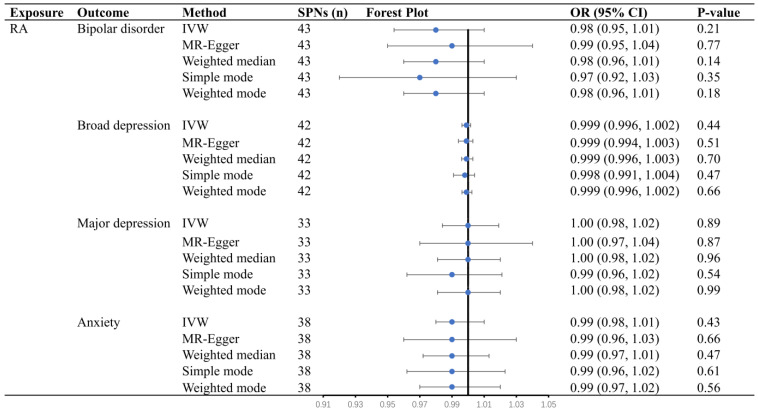
Estimated causal effects between mental illness and rheumatoid arthritis using different MR methods.

Conversely, we estimated the causal effects of RA on mental illness by MR analysis. As a result, there were no causal effects of RA on bipolar disorder (OR = 0.98, 95% CI = 0.96 to 1.01, *p* = 0.21), broad depression (OR = 1.00, 95% CI = 0.996 to 1.002, *p* = 0.44), major depression (OR = 1.00, 95% CI= 0.98 to 1.02, *p* = 0.89), and anxiety (OR = 0.99, 95% CI = 0.98 to 1.01, *p* = 0.43) by the primary method of IVW (Figure 3). Other statistical methods supported this conclusion (*p* > 0.05; Figure 3). 

RA, rheumatoid arthritis; OR, odds ratio; CI, confidence interval; IVW, inverse variance weighted.

The Egger intercept test further suggested no horizontal pleiotropy (*p* of Egger_intercept > 0.05; Table S1). The sensitivity analysis did not find high leverage points with high influence (Figure S2). The result of the MR estimates remained unchanged after the elimination of the outlier using the MR-PRESSO approach (Table S7). After retrieving potential secondary phenotypes for each SNP and excluding SNPs that related to potential confounding factors (Table S5), causal effects in MR analysis have not changed (Table S6).

## 4. Discussion

Based on publicly available summary statistics from different GWAS consortiums, MR analysis showed that bipolar disorder can reduce the risk of RA. On the contrary, RA had no causal effect on bipolar disorder. In addition, there was no bidirectional causal association between broad depression, major depression, anxiety, and RA. However, sensitivity analysis shows that the effect of anxiety on the risk of RA was unstable. 

The MR result allowed us to consider whether there is some kind of related pathological mechanism between bipolar disorder and RA and whether this pathological mechanism has the opposite effect on each other. Research shows that there is a rare mutation in the calcium channel gene in bipolar disorder, resulting in the genetic damage of voltage-gated calcium channels and the increase in calcium signals [50]. At present, the increase in calcium signals may be one of the etiologies of bipolar disorder [51,52]. However, in the pathogenesis of RA, the T cell receptor signal, including the Ca^2+^ signal, is damaged [53]. Increasing intracellular Ca^2+^ concentration can reduce cell viability and interleukin (IL)-6/IL-8/matrix metalloproteinase (MMP)-3 production of RA synovial fibroblasts (RASF) [54], increase IKAROS (encoded by IKZF1) expression [55], and reduce joint inflammation [56]. Therefore, the increase in calcium signals caused by the mutation gene in bipolar disorder patients may be one of the mechanisms of protecting the pathogenesis of RA in patients with bipolar disorder.

The dopamine hypothesis has always been a key theory in the pathophysiology of manic and depressive phases of bipolar disorder. The animal model, the autopsy results of the expression of the dopaminergic gene, and the imaging examinations all proved that bipolar disorder patients had excessive secretion of dopamine, which may be associated with the increase in the density of dopamine transporters and the upregulation of D2/3 receptor levels [57]. Clinical research has also demonstrated the efficacy of antidopaminergic drugs in the treatment of both mania and bipolar depression [57]. Thus, the increase in dopamine secretion may play a role in the pathogenesis of bipolar disorders. On the contrary, clinical evidence suggested that dopamine could effectively inhibit the release of prolactin [58], reduce the activation of synovial macrophages [59], and reduce the inflammatory response [60]. Meanwhile, in RA patients, dopamine could activate the dopaminergic receptor (DR) and inhibit the release of IL-6, IL-8, and tumor necrosis factor (TNF) from synovial fibroblasts [61,62]. It follows that excessive dopamine release may play a role in the mechanism by which bipolar disorder reduces the onset of RA.

In addition, the imbalance of gut microbiota may be a potential mechanism of RA and bipolar disorder. The *Christensenellaceae* was thought a highly heritable taxon in the human gut microbiome [63]. Current studies had found that *Christensenellaceae* could not be detected in patients with bipolar disorder [64], but the relative abundance of *Christensenellaceae* in patients with RA was higher than that of healthy people [65]. The pro-inflammatory or anti-inflammatory effects of *Christensenellaceae* remain undefined. The change in *Christensenellaceae* abundance may be one of the mechanisms underlying the role of bipolar disorder in reducing the risk of RA, but the underlying pathological mechanisms still need further study and in-depth research.

The “inflammatory hypothesis” is considered to be one of the mechanisms leading to the pathogenesis of bipolar disorder [66]. However, one study showed no significant correlation between changes in immune markers in bipolar disorder and autoimmune disease [67]. This means that the mechanism of inflammatory response in RA may be different from that in bipolar disorder. For example, bipolar disorder may be generated through the increasing influx of extracellular calcium through P2X7, N-methyl-D-aspartic acid (NMDA), and L-type calcium channels (LTCCs) and induce an inflammatory response [68]. The autoinflammatory response of RA may be due to genetic variation in human leukocyte antigen (HLA), lymphocyte signaling regulating molecules, or nicotinamide adenine dinucleotide phosphate (NADPH)-oxidase, causing aberrant activation of T and B cells in the body [69]. In addition, limited evidence found that most anti-inflammatory medications do not provide maintenance therapy [70]. Therefore, inflammation may not be the main pathogenesis of bipolar disorder. This may explain why there is no causal relationship between RA and bipolar disorder.

At present, epidemiology mainly supports the increased prevalence of bipolar disorder in patients with RA. The reason for the contradiction between our results and epidemiological findings may be that traditional observational studies were confused by smoking status. The MR analysis showed that smoking was a causal risk factor for bipolar disorder [71] as well as an increased risk for RA [72]. A case-control study found that univariate analysis supported a high incidence of bipolar disorder in patients with RA, but there was no correlation between RA and bipolar disorder after removing the smoking in multivariate analysis [15]. At the same time, the use of medications may also be a confounding factor for the correlation. The study found that patients treated with steroids and opioids were more likely to develop bipolar disorder [73,74]. Since there is no correlation study to rule out the effect of medication use on the results, this may lead to a bias in the results of the correlation study. This result also illustrates that the protective effect of genetically proxied bipolar disorder on RA (reverse causality) does not paradoxically lead to an increased prevalence of bipolar disorder in RA patients.

A large number of epidemiological studies have shown that there was comorbidity between RA, depression, and anxiety [8,23]. However, our study denies the bidirectional causal relationship from the gene perspective. The study’s findings may be supported by clinical research. Methotrexate, leflunomide, hydroxychloroquine, and biological agents are effective prescribed medications for RA treatment. However, clinical research found that the risk of suicidal ideation and anxiety remained or aggravated after the disease activity of RA was decreased by these drugs [75,76]. Conversely, a clinical study of depression in RA patients showed that the disease activity of RA patients did not change significantly after eight weeks of treatment with amitriptyline and paroxetine [77]. A cohort study also showed that indicators of depression and the use of antidepressants were not associated with seropositive RA [78]. However, the results of the MR are unstable. The results showed that anxiety increased the risk of RA after excluding the rs6030245. To date, the reasons for the change in the overall outcome caused by rs6030245 remain unclear and further in-depth research may be required. In addition, increasing the sample size of genetic tools may improve the stability of the results.

There are several advantages to this study. First of all, based on the associated research, this study takes the genetic variable as the design variable, ruled out the interference of residual confounding, and explores the causal relationship between bipolar disorder, depression, anxiety, and RA. Secondly, so far, the incidence of RA in patients with bipolar disorder has not been studied in epidemiology. The analysis results of the protective effect of bipolar disorder on RA obtained in our study may provide a reference for epidemiology in the future. Finally, we found the protective effect of bipolar disorder on RA and speculated that there may be a reverse pathological mechanism between bipolar disorder and RA, especially the effect of the Ca^2+^ signaling pathway and the dopamine concentrations on the pathogenesis of RA, which may open new lines of exciting research.

The study also has its limitations. The conclusion of the causal correlation between anxiety and RA is unstable and increasing the sample size of IVs may lead to more stable results. However, due to the lack of significant loci on anxiety disorders in the literature, we cannot obtain more IVs for research. In addition, most of the genetic variations in this study were heterogeneity in MR analysis. Although we have uniformly used the European population sample to prevent the heterogeneity caused by population stratification, there are many differences among different populations, such as gender, age, social status, and so on, which may still cause differences. Finally, fewer IVs can be used as broad depression after the screening, which may lead to the breadth of the 95% CI. Thus, the results of the causal effect of broad depression on RA may not be accurate.

## Figures and Tables

**Table 1:** Detailed information on the studies and datasets used for Mendelian randomization analyses.

Phenotype	GWAS Reference	Case Ascertainment	Ethnicity	Sample Size
Bipolar disorder	Mullins, N et al., 2021 [32]	Cases were individuals diagnosed with the international consensus criteria for defined bipolar disorder (The Diagnostic and Statistical Manual of Mental Disorders-IVS, International Classification of diseases-9, or International Classification of diseases-10), using structured diagnostic interviews, clinician-managed checklists, or medical history reviews.	European ancestry	41,917 cases and 371,549 controls
Broad depression	Howard, D.M et al., 2018 [33]	Cases were individuals included by self-reported, help-seeking behavior for mental health difficulties.	European ancestry	113,769 cases and 208,811 controls
Major depression	Howard, D.M, et al., 2019 [34]	Cases were ascertained by structured diagnostic interviews, national inpatient electronic records, self-reported major depression symptoms or treatment or electronic records, and self-reported diagnosis or treatment for clinical depression by a medical professional.	European ancestry	246,363 cases and 561,190 controls
Anxiety	Otowa, T et al., 2016 [39]	Cases were individuals diagnosed with anxiety by a psychiatrist. It satisfies the genetic effects shared across the five core anxiety disorders: generalized anxiety disorder (GAD), panic disorder (PD), social phobia, agoraphobia, and specific phobia.	European ancestry	7016 cases and 14,745 controls
RA	Okada, Y et al., 2014 [36]	All cases met the diagnostic criteria of RA of the American Rheumatology Association in 1987 [40] or were diagnosed with RA by experts.	European ancestry	14,361 cases and 43,923 controls

**Table 2:** MR estimates of assessing the causal association between depression, anxiety, and rheumatoid arthritis.

Exposure	Outcome	Method	No. of SNPs	OR (95% CI)	*p*-Value
Broad depression	Rheumatoid arthritis	Inverse variance weighted	2	11.76 (0.23, 600.62)	0.22
Major depression	Rheumatoid arthritis	Inverse variance weighted	60	1.14 (0.87, 1.50)	0.35
		MR-Egger	60	0.66 (0.24, 1.85)	0.44
		Weighted median	60	1.08 (0.76, 1.55)	0.66
		Simple mode	60	0.61 (0.27, 1.35)	0.23
		Weighted mode	60	0.83 (0.49, 1.41)	0.49
Anxiety	Rheumatoid arthritis	Inverse variance weighted	7	1.12 (0.89, 1.40)	0.35
		MR-Egger	7	0.80 (0.19, 3.40)	0.78
		Weighted median	7	1.19 (0.92, 1.55)	0.19
		Simple mode	7	1.16 (0.75, 1.78)	0.52
		Weighted mode	7	1.19 (0.79, 1.78)	0.43

## Data Availability

Data are available upon reasonable request.

## Supplementary Materials

The following supporting information can be downloaded at: https://www.mdpi.com/article/10.3390/jcm12030944/s1. Table S1: Horizontal pleiotropy analysis between exposure factors and outcome variables. Table S2: F statistic between genetic instruments and exposure. Table S3: Potential confounding variables in SNPs associated with bipolar disorder, broad depression, major depression, and anxiety. Table S4: Estimates of causal effect of bipolar disorder, major depression, and anxiety on rheumatoid arthritis after removal of SNPs associated with potential confounders. Table S5: Potential confounding variables in SNPs associated with rheumatoid arthritis. Figure S1: Leave-one-out analysis of associations of genetic risk on mental illness on the risk of rheumatoid arthritis. (a) Bipolar disorder; (b) Broad depression; (c) Major depression; (d) Anxiety.

## Abbreviations

RA, rheumatoid arthritis; MR, Mendelian randomization; GWAS, genome-wide association studies; IVW, inverse variance weighted; OR, odds ratio; PRS, polygenic risk score; SNP, single nucleotide polymorphism; IV, instrumental variable; LD, linkage disequilibrium; MAF, middle allele frequency; GAD, generalized anxiety disorder; PD, panic disorder; CC, case-control; FS, factor scores; SE, standard error; PRESSO, pleiotropy residual sum and outlier; IL, interleukin; MMP, matrix metalloproteinase; DR, dopaminergic receptor; TNF, tumor necrosis factor; NMDA, N-methyl-D-aspartic acid; LTCCs: L-type calcium channels; HLA, human leukocyte antigen; NADPH, nicotinamide adenine dinucleotide phosphate.
