# Supplementary material for: Assessment of Bidirectional Relationships between Mental Illness and Rheumatoid Arthritis: A Two-Sample Mendelian Randomization Study
Source: J Clin Med. 2023 Jan 25;12(3):944. doi: 10.3390/jcm12030944 (PMC9917759; doi:10.3390/jcm12030944)
Supplement: Supplementary file 1 [file jcm-12-00944-s001.zip › jcm-2163922-supplementary.pdf]

Supplementary Material

1 Supplementary Tables

Table S1. Horizontal pleiotropy analysis between exposure factors and outcome variables.

**Exposure**	**Outcome**	**Method**	**P-value**
Bipolar disorder	Rheumatoid arthritis	Cochrane Q statistic	0.01
		MR-Egger regression	0.64
Broad depression			**NA**
Major depression		Cochrane Q statistic	0.01
		MR-Egger regression	0.29
Anxiety		Cochrane Q statistic	0.19
		MR-Egger regression	0.67
Rheumatoid arthritis	Bipolar disorder	Cochrane Q statistic	<0.001
		MR-Egger regression	0.52
	Broad depression	Cochrane Q statistic	0.002
		MR-Egger regression	0.83
	Major depression	Cochrane Q statistic	<0.001
		MR-Egger regression	0.90
	Anxiety	Cochrane Q statistic	0.22
		MR-Egger regression	0.91

Table S2. F statistic between genetic instruments and exposure.

**Exposure**	**Outcome**	**F-statistic (median)**	**F-statistic (minimum)**	**F-statistic (maximum)**
Bipolar disorder	Rheumatoid arthritis	160.76	114.84	288.34
Broad depression		10.19	10.01	10.36
Major depression		90.12	11.37	249.38
Anxiety		144.88	104.86	213.70
Rheumatoid arthritis	Bipolar disorder	322.46	156.98	11546.19
	Broad depression	319.10	156.98	11546.19
	Major depression	315.75	156.98	3732.81
	Anxiety	313.68	156.89	3730.51

Table S3. Potential confounding variables in SNPs associated with bipolar disorder, broad depression, major depression, and anxiety.

Exposure	Outcome	SNP	Potential confounding trait	P-value
Bipolar disorder	Rheumatoid arthritis	rs1487445	Body mass index	3.95e-10
		rs2336147	Body mass index	1.18e-09
		rs4790841	Body mass index	7.49e-19
Broad depression	Rheumatoid arthritis	NA	NA	NA
Major depression	Rheumatoid arthritis	rs34488670	Current tobacco smoking	4.08e-08
		rs3823624	Body mass index	2.53e-13
		rs7685686	Alcohol intake frequency	2.29e-08
		rs7807677	Ever smoked	5.34e-11
		rs2568958	Body mass index in smokers	1.09e-08
		rs301799	Body mass index	6.10E-07
		rs1095626	Body mass index	1.86E-08
		rs10061069	Alcohol intake frequency	1.63E-08
		rs30266	Body mass index	7.95E-06
		rs200949	Ever smoked	7.87E-07
		rs11135349	Body mass index	2.37E-06
Anxiety	Rheumatoid arthritis	rs7528604	Body mass index	2.40e-08

NA: There is no potential confounding variables in SNPs.

Table S4. Estimates of causal effect of bipolar disorder, major depression, and anxiety on rheumatoid arthritis after removal of SNPs associated with potential confounders.

**Exposure**	**Outcome**	**Method**	**No. of SNPs**	**OR (95% CI)**	**P-value**
Bipolar disorder	Rheumatoid arthritis	Inverse variance weighted	30	0.84 (0.72, 0.98)	0.03
		MR Egger	30	0.73 (0.38, 1.39)	0.34
		Weighted median	30	0.85 (0.72, 1.01)	0.06
		Simple mode	30	0.79 (0.54, 1.16)	0.24
		Weighted mode	30	0.81 (0.61, 1.06)	0.13
Major depression	Rheumatoid arthritis	Inverse variance weighted	53	1.12 (0.82, 1.53)	0.48
		MR Egger	53	0.53 (0.13, 2.14)	0.38
		Weighted median	53	1.20 (0.81, 1.78)	0.37

		Simple mode	53	1.42 (0.56, 3.60)	0.46
		Weighted mode	53	0.75 (0.37, 1.53)	0.43
Anxiety	Rheumatoid arthritis	Inverse variance weighted	6	1.10 (0.83, 1.45)	0.52
		MR Egger	6	0.74 (0.14, 3.89)	0.74
		Weighted median	6	1.16 (0.83, 1.61)	0.39
		Simple mode	6	1.13 (0.65, 1.96)	0.68
		Weighted mode	6	1.18 (0.70, 2.00)	0.56

Table S5. Potential confounding variables in SNPs associated with rheumatoid arthritis.

Exposure	Outcome	SNP	Potential confounding trait	P-value
Rheumatoid arthritis	Bipolar disorder	rs12232497	Crohns disease	1.60e-09
		rs12539741	Systemic lupus erythematosus	6.0e-31
		rs17264332	Inflammatory bowel disease	4.79e-08
		rs3087243	Diabetes	2.00e-17
Rheumatoid arthritis	Broad depression	rs12232497	Inflammatory bowel disease	1.02e-17
		rs12539741	Systemic lupus erythematosus	6.0e-31
		rs17208363	Self-reported hypothyroidism or myxoedema	4.51e-11
		rs17264332	Ulcerative colitis	5.12e-11
		rs2229527	Diabetes	2.71e-10
		rs2451258	Psoriasis	3.40e-08
		rs2476601	Self-reported hypothyroidism or myxoedema	3.49e-124
		rs2561477	Self-reported hypothyroidism or myxoedema	3.29e-09
		rs2844456	Self-reported hypothyroidism or myxoedema	7.24e-42
		rs3087243	Self-reported hypothyroidism or myxoedema	8.63e-60
		rs3094633	Diabetes	2.90e-08
		rs3129967	Self-reported hypothyroidism or myxoedema	6.13e-14

		rs34046593	Self-reported hypothyroidism or myxoedema	3.64e-10
		rs34767257	Diabetes	3.10e-15
		rs3749946	Ulcerative colitis	2.00e-15
		rs628357	Diabetes	2.61e-15
		rs706778	Self-reported hypothyroidism or myxoedema	3.48e-13
		rs8032939	Self-reported hypothyroidism or myxoedema	2.23e-11
		rs8192585	Diabetes	1.97e-13
		rs9277550	Self-reported hypothyroidism or myxoedema	1.45e-40
Rheumatoid arthritis	Major depression	rs12232497	Inflammatory bowel disease	1.87e-11
		rs13426947	Self-reported hypothyroidism or myxoedema	5.73e-18
		rs1571878	Self-reported hypothyroidism or myxoedema	1.25e-11
		rs17264332	Ulcerative colitis	5.12e-11
		rs2476601	Self-reported hypothyroidism or myxoedema	3.49e-124
		rs2561477	Self-reported hypothyroidism or myxoedema	3.29e-09
		rs3087243	Self-reported hypothyroidism or myxoedema	8.63e-60
		rs34046593	Self-reported hypothyroidism or myxoedema	3.64e-10

		rs34536443	Self-reported hypothyroidism or myxoedema	6.69e-11
		rs706778	Self-reported hypothyroidism or myxoedema	3.48e-13
		rs8032939	Self-reported hypothyroidism or myxoedema	2.23e-11
Rheumatoid arthritis	Anxiety	rs12232497	Inflammatory bowel disease	1.02e-17
		rs17264332	Ulcerative colitis	5.12e-11
		rs2476601	Diabetes	3.08e-08
		rs3087243	Diabetes	2.30e-17

Table S6. Estimates of causal effect of rheumatoid arthritis on depression, anxiety, and bipolar disorder after removal of SNPs associated with potential confounders.

**Exposure**	**Outcome**	**Method**	**No. of SNPs**	**OR (95% CI)**	**P-value**
Rheumatoid arthritis	Bipolar disorder	Inverse variance weighted	39	0.98 (0.96, 1.01)	0.26
		MR Egger	39	0.99 (0.95, 1.03)	0.61
		Weighted median	39	0.98 (0.96, 1.01)	0.14
		Simple mode	39	0.97 (0.91, 1.02)	0.24
		Weighted mode	39	0.98 (0.96, 1.01)	0.18
Rheumatoid arthritis	Broad depression	Inverse variance weighted	32	1.00 (0.994, 1.002)	0.31
		MR Egger	32	1.00 (0.993, 1.004)	0.58
		Weighted median	32	1.00 (0.995, 1.004)	0.77
		Simple mode	32	1.00 (0.989,1.006)	0.54
		Weighted mode	32	1.00 (0.994, 1.003)	0.69

Rheumatoid arthritis	Major depression	Inverse variance weighted	22	0.99 (0.97, 1.02)	0.66
		MR Egger	22	1.01 (0.92, 1.11)	0.83
		Weighted median	22	0.99 (0.97, 1.02)	0.53
		Simple mode	22	0.98 (0.95, 1.02)	0.43
		Weighted mode	22	0.99 (0.96, 1.02)	0.54
Rheumatoid arthritis	Anxiety	Inverse variance weighted	34	1.00 (0.98, 1.01)	0.61
		MR Egger	34	0.99 (0.91, 1.07)	0.76
		Weighted median	34	0.99 (0.97, 1.01)	0.37
		Simple mode	34	0.99 (0.96, 1.03)	0.72
		Weighted mode	34	0.99 (0.96, 1.03)	0.69

Table S7. Causal effect estimates from MR-PRESSO for the primary analysis.

**Exposure**	**Outcome**	**Method**	**No. of SNPs**	**OR (95% CI)**	**P-value**
Bipolar disorder	Rheumatoid arthritis	MR-PRESSO (raw, 0 outliers)	33	0.82 (0.72, 0.95)	0.007
		MR-PRESSO (outliers corrected, 1 outliers)	32	0.83 (0.72, 0.96)	0.01
Broad depression	Rheumatoid arthritis	MR-PRESSO (raw, 0 outliers)	2	11.76 (0.23, 600.62)	0.22
		MR-PRESSO (outliers corrected, 0 outliers)	2	11.76 (0.23, 600.62)	0.22
Major depression	Rheumatoid arthritis	MR-PRESSO (raw, 0 outliers)	60	1.14 (0.87, 1.50)	0.35
		MR-PRESSO (outliers corrected, 0 outliers)	60	1.14 (0.87, 1.50)	0.35
Anxiety	Rheumatoid arthritis	MR-PRESSO (raw, 0 outliers)	7	1.12 (0.89, 1.40)	0.35

		MR-PRESSO (outliers corrected, 0 outliers)	7	1.12 (0.89, 1.40)	0.35
Rheumatoid arthritis	Bipolar disorder	MR-PRESSO (raw, 0 outliers)	43	0.98 (0.95, 1.01)	0.21
		MR-PRESSO (outliers corrected, 3 outliers)	40	0.98 (0.96, 1.01)	0.26
Rheumatoid arthritis	Broad depression	MR-PRESSO (raw, 0 outliers)	42	1.00 (0.996, 1.002)	0.44
		MR-PRESSO (outliers corrected, 2 outliers)	40	1.00 (0.996, 1.002)	0.40
Rheumatoid arthritis	Major depression	MR-PRESSO (raw, 0 outliers)	33	1.00 (0.98, 1.02)	0.89
		MR-PRESSO (outliers corrected, 0 outliers)	33	1.00 (0.98, 1.02)	0.89
Rheumatoid arthritis	Anxiety	MR-PRESSO (raw, 0 outliers)	38	0.99 (0.98, 1.01)	0.43

	MR-PRESSO (outliers corrected, 0 outliers)	38	0.99 (0.98, 1.01)	0.43

The mr_presso function was run with 10,000 iteration with outler test set to true and a detection threshold of 0.05.

\

Table S8. Estimates of causal effect of anxiety on rheumatoid arthritis after removing rs6030245.

exposure	outcome	method	No. of SNPs	OR (95% CI)	P-value	Heterogeneity
Anxiety	Rheumatoid arthritis	Inverse variance weighted	6	1.27 (1.03, 1.58)	0.03	0.86
		MR Egger	6	1.08 (0.35, 3.39)	0.90	0.76
		Weighted median	6	1.21 (0.92, 1.58)	0.18	NA
		Simple mode	6	1.16 (0.80, 1.66)	0.47	NA
		Weighted mode	6	1.20 (0.86, 1.67)	0.34	NA

Table S9. Heterogeneity analysis between exposure factors and outcome variables

Exposure	Outcome	Method	P-value
Bipolar disorder	Rheumatoid arthritis	Inverse variance weighted	0.002
Broad depression		Inverse variance weighted	1.88E-06
Major depression		Inverse variance weighted	0.008
Anxiety		Inverse variance weighted	0.19
Rheumatoid arthritis	Bipolar disorder	Inverse variance weighted	2.87E-08
	Broad depression	Inverse variance weighted	0.02
	Major depression	Inverse variance weighted	9.06E-05
	Anxiety	Inverse variance weighted	0.23

Table S10. Characteristics of the genetic variants that were used as the instrumental variables for bipolar disorder *

SNP	Beta	SE	EAF	Effect allele	Other allele	P-value
rs2126180	0.0564	0.009	0.457	A	G	1.6E-09
rs10737496	0.0545	0.009	0.444	C	T	7.2E-09
rs4619651	0.0658	0.010	0.670	G	A	4.8E-11
rs17183814	0.1026	0.019	0.924	G	A	0.000000027
rs13417268	0.0620	0.011	0.758	C	G	0.000000021
rs2011302	0.0535	0.010	0.377	A	T	0.000000043
rs2719164	0.0516	0.010	0.564	A	G	0.000000049
rs9834970	0.0834	0.009	0.481	C	G	6.6E-19
rs2336147	0.0677	0.009	0.498	T	C	3.6E-13
rs115694474	0.0658	0.012	0.799	T	A	0.000000024
rs696366	0.0516	0.009	0.550	C	A	0.000000045
rs112481526	0.0630	0.011	0.256	G	A	1.9E-09
rs28565152	0.0677	0.011	0.238	A	G	0.000000002
rs6865469	0.0583	0.010	0.274	T	G	0.000000017
rs6887473	0.0602	0.011	0.739	G	A	8.8E-09
rs10043984	0.0602	0.011	0.236	T	C	0.000000037
rs10866641	0.0630	0.009	0.575	T	C	2.8E-11
rs13195402	0.1363	0.018	0.919	G	T	5.8E-15

rs1487445	0.0751	0.009	0.487	T	C	1.5E-15
rs4331993	0.0545	0.010	0.382	A	T	0.00000002
rs10455979	0.0554	0.010	0.500	G	C	4.2E-09
rs12668848	0.0573	0.010	0.575	G	A	1.9E-09
rs113779084	0.0760	0.010	0.299	A	G	1.4E-13
rs6954854	0.0583	0.009	0.425	G	A	5.9E-10
rs12672003	0.0917	0.016	0.113	G	A	2.7E-09
rs11764361	0.0611	0.010	0.668	A	G	3.5E-09
rs6946056	0.0535	0.010	0.623	C	A	0.000000037
rs10255167	0.0658	0.012	0.778	A	G	0.000000016
rs62489493	0.0898	0.014	0.128	G	C	2.6E-11
rs3088186	0.0564	0.010	0.287	T	C	0.000000021
rs2953928	0.1169	0.020	0.067	A	G	6.3E-09
rs6992333	0.0602	0.010	0.410	G	A	1.6E-09
rs10973201	0.0962	0.017	0.110	C	T	0.000000025
rs62581014	0.0649	0.012	0.366	T	C	0.000000028
rs1998820	0.0834	0.015	0.886	T	A	0.000000041
rs10994415	0.1178	0.017	0.082	C	T	1.1E-11
rs10761661	0.0516	0.009	0.472	T	C	0.000000047
rs2273738	0.0917	0.014	0.135	T	C	1.6E-11

rs174592	0.0714	0.010	0.395	G	A	9.9E-14
rs4672	0.1017	0.017	0.083	A	G	3.4E-09
rs475805	0.0677	0.011	0.767	A	G	0.000000002
rs678397	0.0545	0.009	0.457	T	C	5.5E-09
rs12575685	0.0649	0.010	0.327	A	G	1.2E-10
rs12289486	0.0825	0.015	0.115	T	C	0.000000033
rs11062170	0.0779	0.010	0.333	C	G	1.9E-15
rs35306827	0.0658	0.011	0.775	G	A	3.6E-09
rs2693698	0.0535	0.009	0.551	G	A	0.00000002
rs35958438	0.0639	0.012	0.772	G	A	0.000000038
rs4447398	0.0825	0.014	0.131	A	C	2.6E-09
rs62011709	0.0620	0.011	0.747	T	A	0.000000014
rs748455	0.0677	0.010	0.719	T	C	5E-11
rs4702	0.0573	0.010	0.446	G	A	3.5E-09
rs28455634	0.0630	0.010	0.620	G	A	2.6E-10
rs7199910	0.0554	0.010	0.312	G	T	0.000000017
rs12932628	0.0564	0.010	0.487	T	G	6.7E-09
rs4790841	0.0723	0.013	0.151	T	C	0.000000031
rs11870683	0.0573	0.01	0.650	T	A	0.000000028
rs61554907	0.0871	0.015	0.124	T	G	0.000000016

rs228768	0.0649	0.01	0.294	G	T	2.8E-10
rs67712855	0.0677	0.01	0.687	T	G	4.2E-11
rs6032110	0.0573	0.009	0.512	A	G	0.000000001
rs237460	0.0554	0.009	0.412	T	C	4.3E-09
rs13044225	0.0545	0.01	0.440	G	A	8.5E-09
rs5758064	0.0526	0.009	0.523	T	C	0.00000002

SNP, single nucleotide polymorphism; SE, standard error; EAF, effect allele frequency; Beta coefficients are in standard deviation (SD) unit per allele; effect allele is the bipolar disorder-raising allele.

*Source: Mullins, N et al, 2021 [1]

Table S11. Characteristics of the genetic variants that were used as the instrumental variables for broad depression*

SNP	Beta	SE	EAF	Effect allele	Other allele	P-value
rs3807865	0.0082	0.0012	0.4117	A	G	7.28E-12
rs10501696	-0.0070	0.0012	0.4987	G	A	6.73E-11

SNP, single nucleotide polymorphism; SE, standard error; EAF, effect allele frequency; Beta coefficients are in standard deviation (SD) unit per allele; effect allele is the broad depression-raising allele.

*Source: Howard, D.M et al, 2018 [2]

Table S12. Characteristics of the genetic variants that were used as the instrumental variables for major depression*

SNP	Beta	SE	EAF	Effect allele	Other allele	P-value
rs301799	-0.0254	0.0022	0.5694	T	C	4.05E-31
rs1002656	-0.0210	0.0024	0.7033	T	C	4.84E-19
rs1466887	-0.0125	0.0022	0.5511	T	C	1.37E-08
rs11579246	0.0320	0.0037	0.9067	A	G	8.07E-18
rs1890946	-0.0194	0.0022	0.4671	T	C	8.62E-19
rs10789214	0.0137	0.0022	0.5661	T	C	3.81E-10
rs2568958	0.0336	0.0022	0.6156	A	G	4.45E-52
rs10890020	-0.0267	0.0022	0.5156	A	G	3.31E-34
rs113188507	0.0234	0.0025	0.2838	A	G	2.94E-21
rs10913112	-0.0214	0.0023	0.3767	T	C	1.72E-20
rs72710803	-0.0247	0.0038	0.9121	A	C	1.35E-10
rs169235	-0.0170	0.0025	0.7530	A	G	1.61E-11
rs17641524	-0.0243	0.0027	0.2091	T	C	7.9E-20
rs12052908	-0.0211	0.0022	0.5325	A	T	3.98E-22
rs1568452	0.0293	0.0022	0.3851	T	C	3.08E-40
rs7585722	-0.0215	0.0030	0.8458	T	C	5.47E-13
rs1226412	0.0232	0.0027	0.7917	T	C	3.35E-18
rs62188629	0.0169	0.0024	0.3136	A	G	6.15E-13

rs4346585	-0.0163	0.0024	0.6960	T	C	4.87E-12
rs13084037	-0.0217	0.0025	0.7740	A	G	1.79E-17
rs7624336	0.0092	0.0027	0.2087	T	G	0.000548
rs141954845	0.0179	0.0022	0.3880	A	G	1.18E-15
rs6783233	0.0177	0.0024	0.2833	T	C	1.14E-13
rs1095626	-0.0285	0.0022	0.5799	T	C	6.31E-39
rs7685686	0.0173	0.0022	0.5753	A	G	5.52E-15
rs34937911	0.0244	0.0034	0.8838	T	C	5.39E-13
rs45510091	0.0463	0.0049	0.9472	A	G	7.74E-21
rs35553410	-0.0159	0.0025	0.7462	T	C	2.18E-10
rs7659414	-0.0089	0.0022	0.5782	A	C	4.32E-05
rs60157091	0.0205	0.0022	0.515	T	C	5.54E-21
rs3099439	-0.0208	0.0022	0.5288	T	C	1.61E-21
rs10061069	-0.0250	0.0026	0.2212	C	G	5.17E-22
rs30266	0.0323	0.0023	0.3296	A	G	1.99E-45
rs11135349	-0.025	0.0022	0.4713	A	C	2.18E-30
rs200949	0.0433	0.0034	0.8744	A	G	2.13E-37
rs9363467	0.0158	0.0023	0.6035	T	C	2.5E-12
rs7758630	-0.0165	0.0022	0.4051	A	T	7.79E-14
rs1933802	-0.0198	0.0022	0.4536	C	G	1.36E-19

rs2876520	-0.0187	0.0022	0.5271	C	G	2.65E-17
rs725616	0.0135	0.0022	0.3644	T	C	1.06E-09
rs2029865	-0.0184	0.0022	0.4534	A	T	4.02E-17
rs3823624	0.0294	0.0028	0.8067	T	C	1.61E-26
rs2043539	0.0220	0.0022	0.4177	A	G	8.32E-24
rs2247523	-0.0159	0.0021	0.5319	C	G	9.05E-14
rs16887442	0.0148	0.0022	0.4347	T	C	1.25E-11
rs58104186	0.0190	0.0022	0.4689	A	G	3.56E-18
rs7807677	0.0211	0.0022	0.5505	T	C	5.4E-22
rs7837935	-0.0226	0.0031	0.1522	T	G	1.15E-13
rs67436663	-0.0141	0.0025	0.2402	C	G	2.83E-08
rs1354115	0.0186	0.0022	0.6243	A	C	4.39E-17
rs1982277	0.0206	0.0026	0.7594	T	C	1.28E-15
rs263645	0.0176	0.0022	0.5438	A	T	6.92E-16
rs3793577	-0.0211	0.0022	0.4665	A	G	5.58E-22
rs59283172	-0.0174	0.0036	0.1069	A	G	1.18E-06
rs34653192	-0.0196	0.0024	0.3196	C	G	3.39E-16
rs7030813	0.0251	0.0022	0.3736	T	C	8.88E-30
rs10817969	0.0169	0.0024	0.7173	T	G	1.32E-12
rs913930	-0.0219	0.0023	0.6433	A	G	1.03E-21

rs2670139	-0.0177	0.0025	0.7609	T	C	2.19E-12
rs997934	0.0163	0.0023	0.3795	T	C	4.6E-13
rs1021363	0.0226	0.0023	0.3547	A	G	3.77E-23
rs1448938	0.0172	0.0022	0.4171	A	G	3.74E-15
rs2509805	0.0191	0.0024	0.3209	T	C	4.92E-16
rs198457	-0.0213	0.0028	0.1925	T	C	5.41E-14
rs58621819	-0.0169	0.0027	0.7903	A	T	2.17E-10
rs7117514	-0.0166	0.0022	0.5417	A	G	3.64E-14
rs7932640	0.0227	0.0022	0.4417	T	C	3.4E-25
rs61902811	-0.0289	0.0022	0.3682	A	G	5.17E-39
rs2187490	-0.0152	0.0039	0.9106	T	G	8.49E-05
rs57344483	-0.0241	0.0041	0.9259	A	G	5.26E-09
rs78337797	0.0229	0.0034	0.8781	T	G	1.37E-11
rs56314503	-0.0203	0.0025	0.7487	T	G	1.28E-16
rs10774600	-0.0133	0.0030	0.1656	T	C	1.07E-05
rs3213572	0.0196	0.0021	0.4745	A	G	5.51E-20
rs1409379	0.0164	0.0025	0.7641	T	C	8.26E-11
rs1343605	0.0231	0.0022	0.3840	A	C	1.62E-25
rs9592461	0.0243	0.0022	0.4874	A	G	1.17E-28
rs9545360	-0.0167	0.0029	0.1807	A	C	6.7E-09

rs4772087	0.0209	0.0023	0.3732	T	C	2.34E-20
rs61990288	-0.0249	0.0021	0.5083	A	G	1.96E-31
rs1956373	-0.0169	0.0025	0.7436	T	G	6.08E-12
rs1152578	-0.0152	0.0022	0.4357	T	C	4.19E-12
rs1045430	-0.0226	0.0022	0.4792	T	G	4.44E-25
rs10149470	-0.0206	0.0021	0.4869	A	G	6.88E-22
rs8037355	-0.0192	0.0022	0.5556	T	C	1.52E-18
rs34488670	-0.0193	0.0027	0.7887	T	C	4.69E-13
rs7193263	-0.0206	0.0023	0.6679	A	G	3.83E-19
rs7198928	0.0219	0.0022	0.6159	T	C	3.71E-23
rs7200826	0.027	0.0025	0.2551	T	C	3.09E-28
rs56887639	-0.0171	0.0024	0.7264	A	G	6.04E-13
rs12923444	-0.0237	0.0023	0.5625	A	C	1.76E-24
rs75581564	0.0248	0.0034	0.1165	A	G	3.49E-13
rs12967855	0.0207	0.0023	0.3295	A	G	1.32E-19
rs7227069	0.0242	0.0022	0.4326	A	G	2.13E-28
rs62091461	-0.0199	0.0026	0.2274	T	C	1.75E-14
rs12966052	-0.0187	0.0028	0.1805	C	G	3.76E-11
rs12967143	-0.0256	0.0024	0.6984	C	G	1.61E-27
rs7241572	0.02	0.0027	0.2010	A	G	2.56E-13

rs33431	0.0121	0.0022	0.6144	T	C	4.69E-08
rs143186028	0.0244	0.0028	0.1778	T	G	7.23E-18
rs12624433	0.0191	0.0025	0.2584	A	G	2.16E-14
rs5995992	-0.0301	0.0024	0.7155	T	C	2.6E-35

SNP, single nucleotide polymorphism; SE, standard error; EAF, effect allele frequency; Beta coefficients are in standard deviation (SD) unit per allele; effect allele is the major depression-raising allele.

*Source: Howard, D.M, et al, 2019 [3]

Table S13. Characteristics of the genetic variants that were used as the instrumental variables for anxiety*

SNP	Beta	SE	EAF	Effect allele	Other allele	P-value
rs7528604	-0.1165	0.0174	0.385	A	G	5.39E-11
rs1458103	-0.1076	0.0194	0.741	A	C	6.19E-08
rs113209956	-0.1887	0.0353	0.085	T	C	6.36E-08
rs6462203	-0.1043	0.0194	0.265	A	C	1.09E-07
rs6030245	0.1133	0.0222	0.795	T	C	5.06E-07
rs11855560	0.0853	0.0172	0.469	T	C	6.96E-07
rs2451828	0.2927	0.0592	0.019	T	C	7.37E-07
rs16916239	-0.1020	0.0211	0.783	A	G	8.96E-07
rs79928194	-0.1485	0.0305	0.905	T	C	1.26E-06
rs342422	-0.0834	0.0224	0.530	A	G	1.28E-06

SNP, single nucleotide polymorphism; SE, standard error; EAF, effect allele frequency; Beta coefficients are in standard deviation (SD) unit per allele; effect allele is the anxiety -raising allele.

*Source: Otowa, T et al, 2016 [4]

Table S14. Characteristics of the genetic variants that were used as the instrumental variables for rheumatoid arthritis*

SNP	Beta	SE	EAF	Effect allele	Other allele	P-value
rs61828284	-0.19845	0.034487	0.9205	T	C	8.70E-09
rs28411352	0.10436	0.017868	0.7495	T	C	5.20E-09
rs2476601	-0.59333	0.022782	0.0944	G	A	1.60E-149
rs2240336	-0.10536	0.017399	0.4145	T	C	1.40E-09
rs60733400	-0.10536	0.017365	0.3141	A	G	1.30E-09
rs9261602	0.18633	0.021503	0.5477	G	A	4.50E-18
rs13426947	0.131028	0.018694	0.1909	A	G	2.40E-12
rs3087243	-0.13926	0.015307	0.4702	A	G	9.20E-20
rs34695944	0.116534	0.015438	0.3608	C	T	4.40E-14
rs2661798	0.094311	0.015476	0.5517	T	A	1.10E-09
rs9653442	-0.10536	0.015155	0.4533	T	C	3.60E-12
rs4452313	0.105361	0.016684	0.6918	T	A	2.70E-10
rs9310852	0.083382	0.015078	0.5378	G	A	3.20E-08
rs73081554	0.165514	0.030301	0.9483	T	C	4.70E-08
rs34046593	0.139762	0.016809	0.3211	A	G	9.20E-17
rs2561477	-0.10536	0.016958	0.3022	A	G	5.20E-10

rs7731626	-0.19845	0.020177	0.3777	A	G	7.90E-23
rs212389	0.09531	0.01564	0.3738	A	G	1.10E-09
rs9258570	-0.14842	0.020383	0.1988	C	G	3.30E-13
rs17264332	0.162519	0.018316	0.831	G	A	7.10E-19
rs9275183	0.776529	0.022961	0.836	G	A	1.00E-200
rs13196363	-0.27444	0.026895	0.8121	T	C	1.90E-24
rs2844456	0.891598	0.029191	0.9602	C	T	1.00E-200
rs1042153	-0.37106	0.020927	0.2296	A	G	2.40E-70
rs34431565	-0.44629	0.043257	0.0368	T	G	5.90E-25
rs1571878	-0.11653	0.014884	0.4215	T	C	4.90E-15
rs9368695	-0.35668	0.063123	0.9881	T	C	1.60E-08
rs2233434	0.287682	0.052072	0.0497	G	A	3.30E-08
rs12539741	0.157004	0.023156	0.8996	T	C	1.20E-11
rs11574914	0.122218	0.016545	0.6849	A	G	1.50E-13
rs10985070	-0.08338	0.014784	0.5656	A	C	1.70E-08
rs12764378	0.131028	0.017813	0.2445	A	G	1.90E-13
rs537544	-0.11653	0.017927	0.6203	T	C	8.00E-11
rs706778	0.10436	0.015223	0.4135	T	C	7.10E-12
rs10790268	0.162519	0.020627	0.1889	G	A	3.30E-15
rs9603608	-0.10436	0.016035	0.6561	C	A	7.60E-11

rs8032939	0.116534	0.016626	0.2416	C	T	2.40E-12
rs8026898	0.14842	0.017518	0.2853	A	G	2.40E-17
rs13330176	0.113329	0.019714	0.2396	A	T	9.00E-09
rs12232497	0.094311	0.015981	0.4722	C	T	3.60E-09
rs592390	-0.09531	0.016174	0.5229	C	T	3.80E-09
rs34536443	-0.37844	0.046596	0.0288	C	G	4.60E-16
rs4239702	0.139262	0.018434	0.7127	C	T	4.20E-14
rs8133843	0.09531	0.016385	0.6252	A	G	6.00E-09
rs225433	-0.12783	0.022705	0.8111	G	C	1.80E-08
rs2069235	0.10436	0.016568	0.2992	A	G	3.00E-10

SNP, single nucleotide polymorphism; SE, standard error; EAF, effect allele frequency; Beta coefficients are in standard deviation (SD) unit per allele; effect allele is rheumatoid arthritis -raising allele.

*Source: Okada, Y et al, 2014 [5]

Reference:

1. Mullins, N.; Forstner, A.J.; O'Connell, K.S.; Coombes, B.; Coleman JR, I.; Qiao, Z.; Als, T.D.; Bigdeli, T.B.; Børte, S.; Bryois, J.; et al. Genome-wide association study of more than 40,000 bipolar disorder cases provides new insights into the underlying biology. Nat. Genet. 2021, 53, 817–829.

2. Howard, D.M.; Adams, M.J.; Shirali, M.; Clarke, T.K.; Marioni, R.E.; Davies, G.; Coleman JR, I.; Alloza, C.; Shen, X.; Barbu, M.C.; et al. Genome-wide association study of depression phenotypes in uk biobank identifies variants in excitatory synaptic pathways. Nat. Commun. 2018, 9, 1470.

3. Howard, D.M.; Adams, M.J.; Clarke, T.K.; Hafferty, J.D.; Gibson, J.; Shirali, M.; Coleman JR, I.; Hagenaars, S.P.; Ward, J.; Wigmore, E.M.; et al. Genome-wide meta-analysis of depression identifies 102 independent variants and highlights the importance of the prefrontal brain regions. Nat. Neurosci. 2019, 22, 343–352.

4. Otowa, T.; Hek, K.; Lee, M.; Byrne, E.M.; Mirza, S.S.; Nivard, M.G.; Bigdeli, T.; Aggen, S.H.; Adkins, D.; Wolen, A.; et al. Meta-analysis of genome-wide association studies of anxiety disorders. Mol. Psychiatry 2016, 21, 1391–1399.

5. Okada, Y.; Wu, D.; Trynka, G.; Raj, T.; Terao, C.; Ikari, K.; Kochi, Y.; Ohmura, K.; Suzuki, A.; Yoshida, S.; et al. Genetics of rheumatoid arthritis contributes to biology and drug discovery. Nature 2014, 506, 376–381.

2 Supplementary Figures

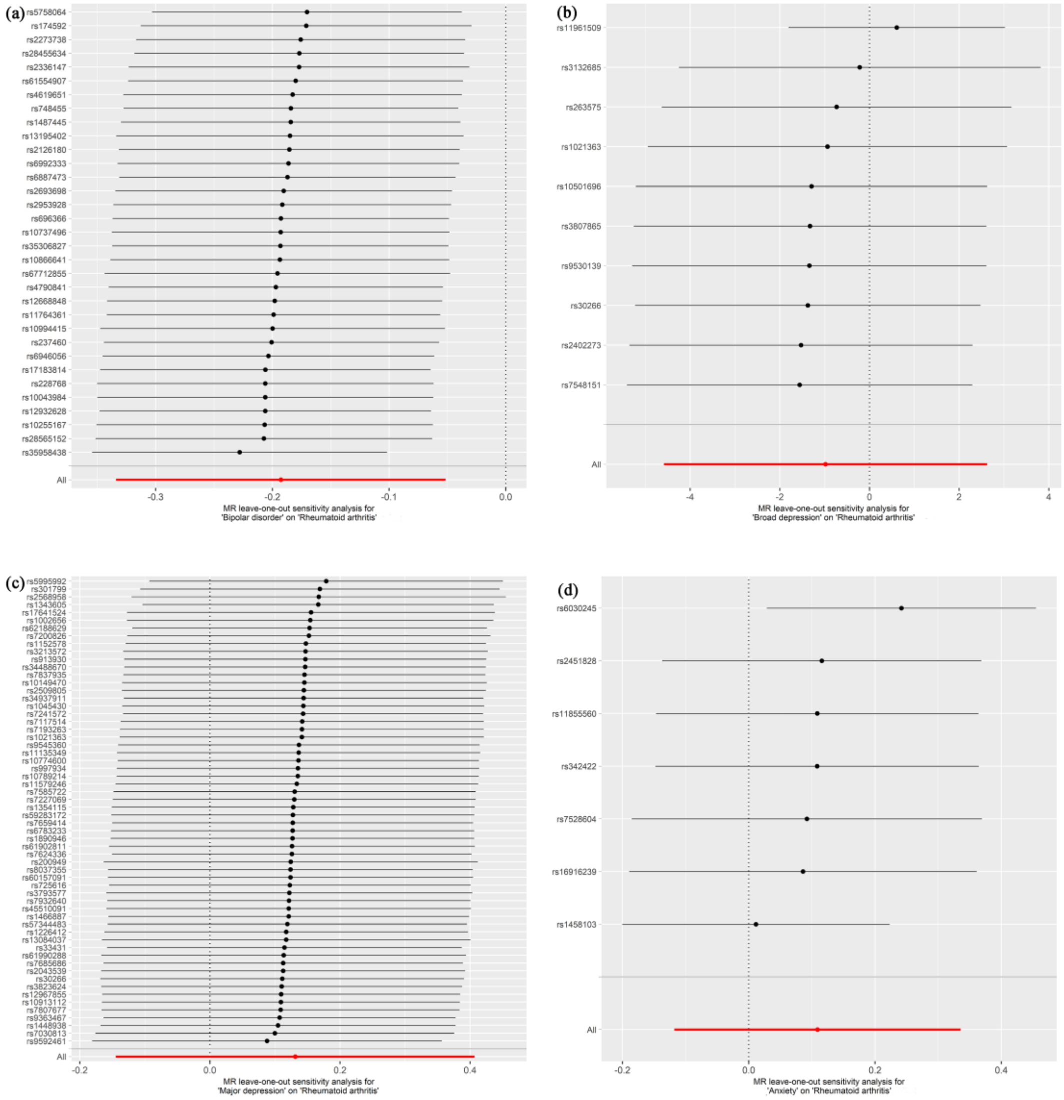

Figure S1. Leave-one-out analysis of associations of genetic risk on mental illness on the risk of rheumatoid arthritis. (a) Bipolar disorder; (b) Broad depression; (c) Major depression; (d) Anxiety.

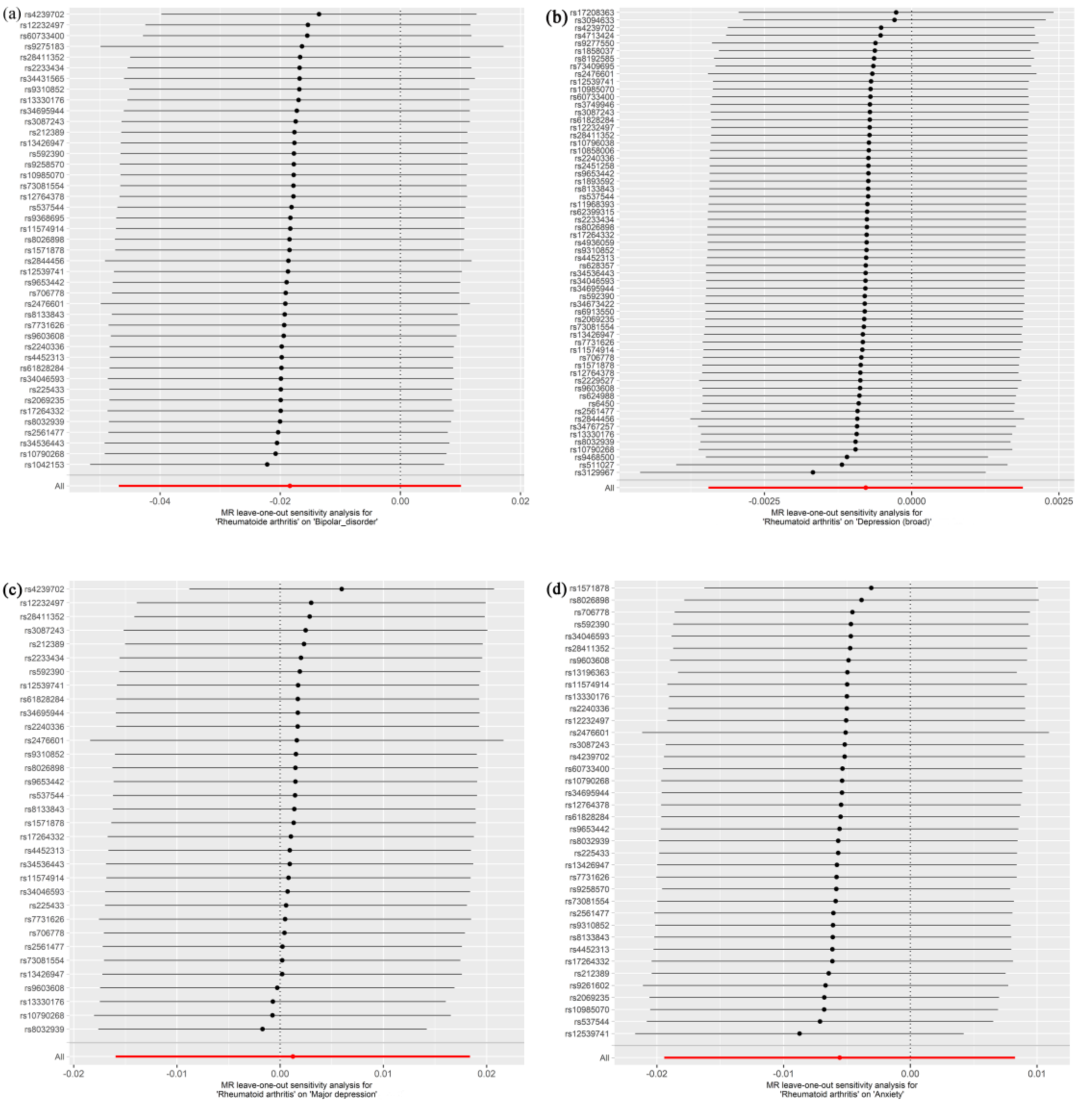

Figure S2. Leave-one-out analysis of associations of genetic risk on rheumatoid arthritis on the risk of mental illness. (a) Bipolar disorder; (b) Broad depression; (c) Major depression; (d) Anxiety.
